# Feasibility and challenges of inpatient psychotherapy for psychosis: lessons learned from a veterans health administration pilot randomized controlled trial

**DOI:** 10.1186/s13104-016-2179-z

**Published:** 2016-07-30

**Authors:** Matthew Tyler Boden, Brandon A. Gaudiano, Robyn D. Walser, Christine Timko, William Faustman, Sarah Yasmin, Ruth C. Cronkite, Marcel O. Bonn-Miller, John F. McCarthy

**Affiliations:** 1 Center for Innovation to Implementation, VA Palo Alto Health Care System, 795 Willow Road (152-MPD), Menlo Park, CA 94025 USA; 2 Department of Psychiatry and Human Behavior, Brown Medical School, Providence, RI USA; 3 Psychosocial Research Program, Butler Hospital, Providence, RI USA; 4 National Center for PTSD, VA Palo Alto Healthcare System, Palo Alto, CA USA; 5 University of California, Berkeley, CA USA; 6 Palo Alto Division, VA Palo Alto Healthcare System, Palo Alto, CA USA; 7 Department of Psychiatry and Behavioral Sciences, Stanford University School of Medicine, Stanford, CA USA; 8 Department of Sociology, Stanford University, Stanford, CA USA; 9 Center for Primary Care and Outcomes Research, Stanford University School of Medicine, Stanford, CA USA; 10 Center of Excellence in Substance Abuse Treatment and Education, Philadelphia VA Medical Center, Philadelphia, PA USA; 11 Serious Mental Illness Treatment Resource and Evaluation Center, VA Office of Mental Health Operations, Ann Arbor, MI USA; 12 Department of Psychiatry, University of Michigan, Ann Arbor, MI USA

**Keywords:** Randomized controlled trial, Hybrid type I, Acceptance and commitment therapy, Feasibility, Psychosis

## Abstract

**Background:**

In large health care systems, decision regarding broad implementation of psychotherapies for inpatients with psychosis require substantial evidence regarding effectiveness and feasibility for implementation. It is important to recognize challenges in conducting research to inform such decisions, including difficulties in obtaining consent from and engaging inpatients with psychosis in research. We set out to conduct a feasibility and effectiveness Hybrid Type I pilot randomized controlled trial of acceptance and commitment therapy (ACT) and a semi-formative evaluation of barriers and facilitators to implementation.

**Findings:**

We developed a training protocol and refined an ACT treatment manual for inpatient treatment of psychosis for use at the Veterans Health Administration. While our findings on feasibility were mixed, we obtained supportive evidence of the acceptability and safety of ACT. Identified strengths of ACT included a focus on achievement of valued goals rather than symptoms. Weaknesses included that symptoms may limit patient’s understanding of ACT. Facilitators included building trust and multi-stage informed consent processes. Barriers included restrictive eligibility criteria, rigid use of a manualized protocol, and individual therapy format. Conclusions are limited by our randomization of only 18 patient participants (with nine completing all aspects of the study) out of 80 planned.

**Conclusions:**

Future studies should include (1) multi-stage informed consent processes to build trust and alleviate patient fears, (2) relaxation of restrictions associated with obtaining efficacy/effectiveness data, and (3) use of Hybrid Type II and III designs.

## Background

Psychotic disorders, which include schizophrenia, schizoaffective disorder, and bipolar disorder with psychosis, are complex and often chronic conditions. Health systems seek to deliver ongoing outpatient care to help patients maintain function and symptom stability. However, this is difficult due to periodic exacerbations [5], low tolerance of normative stressors [17], and disengagement from mental health treatment [15]. Indeed, patients with psychotic disorders receive substantial and costly inpatient treatment [4, 13]. To help patients achieve stability and return to the community, recommended inpatient treatments include a mix of medication, psychotherapy, and engagement with other patients and staff [7]. Unfortunately, patients with psychosis are often readmitted to inpatient units, suggesting the need for recovery-focused inpatient treatment approaches that contribute to lasting gains.

Empirically supported psychotherapies such as acceptance and commitment therapy (ACT) are more recovery-oriented than symptom-focused [11]. ACT utilizes mindfulness and acceptance strategies to increase patients’ abilities to cope with symptoms and dysfunction [11]. Two randomized controlled trials (RCTs) have demonstrated the effectiveness of ACT relative to usual care: Patients receiving ACT demonstrate greater improvements in symptoms and coping, and reduced re-hospitalization rates post-treatment [3, 9].

Broad implementation of treatment for inpatients with psychosis, and specifically of psychotherapies such as ACT, typically requires substantial evidence regarding effectiveness and feasibility of implementation. Such evidence is typically garnered from research studies. This is especially true within large health care systems, such as the Veterans Health Administration (VHA). The two prior RCTs demonstrating the effectiveness of ACT were conducted in non-VHA settings. Yet, unique characteristics (e.g., structure, patients served) of large health care systems, such as the VHA may influence implementation and effectiveness of ACT. Thus, it is important to recognize challenges involved in the process of developing such information. Notably, it is difficult to obtain consent and engage psychiatric inpatients with psychosis in research (e.g., [14]).

We set out to conduct a feasibility/effectiveness Hybrid Type I study [6] incorporating a pilot RCT of ACT and a semi-formative evaluation of barriers and facilitators to future implementation of ACT for inpatients diagnosed with psychosis. Thus, we focused on obtaining data regarding feasibility and implementation of ACT with this patient population, and secondarily gaining preliminary information regarding ACT’s effectiveness (reported at ClinicalTrials.gov: NCT01981356). Our focus was consistent with the stage model by the National Institutes of Health [16] and with critiques of the stage model that emphasize expediency early in the treatment development process [6].

The study had two primary aims: (1) to assess whether ACT demonstrates feasibility, acceptability, and safety within the VHA inpatient setting, and (2) to obtain data from inpatient unit staff regarding system-, clinician-, and patient-level barriers and facilitators to providing and implementing ACT among veterans with psychosis, as guided by the reach, effectiveness, adoption, implementation, maintenance (RE-AIM ) framework [10].

## Methods

### Participants

We recruited two sets of participants: (1) 29 VHA inpatients with current or recent (within past week) psychotic symptoms (hallucinations and/or delusions) related to a DSM-IV-R [1] diagnosed psychotic or mood (but not substance use) disorder, and (2) four inpatient psychiatry unit staff members. Study or inpatient unit staff approached patient participants, briefly described the study, and elicited interest. Likewise, study staff approached inpatient unit staff.

### Treatments

We randomly assigned patient participants to either treatment as usual (TAU) plus four individual sessions of ACT (adapted from [9]), or TAU during their entire length of stay (*M* = 18.4, *SD* = 13.1), with the addition of four sessions to equate for staff time and attention.

### Measures

#### Patients

We collected data from patients immediately prior to treatment initiation and immediately prior to discharge.

Measures of feasibility, acceptability, and safety included: (a) the ability to recruit and consent two eligible patient participants per week (for 40 weeks) to be randomized to TAU+ACT or TAU; (b) patient attendance at three out of four ACT sessions, on average; (c) patient and therapist reported treatment satisfaction (adapted Client Satisfaction Questionnaire-8; [2]) and therapeutic alliance (Working Alliance Inventory; [12]); and (d) the occurrence of zero serious adverse events.

#### Staff

We conducted 30–60 min semi-structured interviews using the RE-AIM (reach, efficacy, adoption, implementation and maintenance) framework [10] and the RE-AIM Planning Tool [8] to identify barriers and facilitators to patient participation in ACT and implementation of ACT, broadly, and how to address them, and perceptions about ACT effectiveness in achieving desired outcomes. We coded interviews as guided by the rapid analysis methodology of Curran et al. [6].

### Findings

#### Aim 1

Findings regarding feasibility and acceptability of ACT were mixed. We fell short of meeting our initial goal of recruiting and randomizing two eligible participants per week for a total of 80 participants. We reviewed charts for 429 admission patients over 8 months; we approached 67 patients who potentially met study criteria. Twenty-nine patients provided informed consent and agreed to a comprehensive assessment for eligibility, yielding 18 eligible participants (all were male, average age was 53.4 (*SD* = 17.5), 38.5 % were Caucasian, 30.8 % were Hispanic/Latino, 23.1 % were Black/African–American, and 7.7 % were Asian/Asian–American). Twelve were randomized to ACT+TAU and six to TAU; however data could not be utilized for two participants (one signed an invalid consent and another was on a legal hold when consented). One additional participant in each condition withdrew, and five participants did not complete the post-treatment assessment due to discharging themselves or being discharged early and without notice, leaving nine participants (ACT+TAU = 5; TAU = 4) who completed all aspects of the study. We enrolled four of six planned inpatient psychiatry unit staff (one nursing assistant, one nurse, two psychologists, 75 % were male) to assess barriers and facilitators.

For those receiving treatment, we successfully met our aims related to treatment acceptability, and safety. Intent-to-treat participants assigned to ACT+TAU attended 2.8 sessions on average (*SD* = 1.6), with five completing all four sessions. Participants completing ACT+TAU reported moderate-to-high levels of ACT satisfaction (*M* = 2.9 of 4, *SD* = 0.3) and alliance (*M* = 4.7 of 7, *SD* = 1.0). ACT+TAU was safe, as indicated by no serious adverse events.

#### Aim 2

Staff members were consistent in their reports of (a) strengths of ACT (patient with chronic and pervasive psychosis benefit from focus on achievement of valued goals rather than symptom reduction); (b) weaknesses of ACT (symptoms [e.g., paranoia] and related sequela [e.g., cognitive impairment] may limit patients’ understanding of ACT); (c) facilitators to implementation (build trust with patients before treatment, offer ACT in group format; be flexible in application of manual; engage in more experiential or “hands-on” exercises; utilize a team-based approach to applying ACT); and (d) barriers to patient participation and implementation (difficult to obtain supporting research evidence due to restrictive eligibility criteria, rigid application of manualized treatment, patients’ limited time and motivation given their many activities and appointments).

## Discussion

We encountered important challenges related to conducting research and implementing psychotherapy on acute psychiatry inpatient units. The most notable challenge concerned patient recruitment. Indeed, our recruitment and randomization of 18 patient participants, with only nine participants completing all aspects of the study, substantially limited our ability to draw conclusions regarding ACT. Identified as a weakness of ACT and barrier to implementation in our qualitative interviews, patient characteristics may have hindered recruitment and engagement in ACT [15]. Additionally, numerous patients we approached were involuntarily committed and conserved and thus ineligible to provide informed consent. Others were unwilling to provide consent, or provided consent and later withdrew or were discharged, often without notice. These issues reflect inherent difficulties in conducting research with psychiatry inpatients [14].

For future studies, we recommend use of a multi-stage informed consent processes to build trust and alleviate patients’ potential fears. We identified building trust as a potential facilitator to implementation that may increase enrollment and enhance the therapeutic process itself. However, we noted the difficulty of building trust when conducting a psychotherapy RCT on an inpatient unit, as time constraints limit interactions with patients. For example, the informed consent process, pre- and post-treatment assessments, and four treatment sessions for our proposed trial needed to take place within a given patient’s relatively short inpatient stay. Thus, the informed consent/building of trust process might supplant some of the therapy itself. In VHA settings, treatment could potentially continue post-discharge, since electronic medical records allow for tracking of patients.

Aspects of the treatment and its implementation presented further challenges to conducting the trial. Restrictive eligibility criteria (e.g., excluding patients with substance-induced psychosis) and rigid application of a manualized protocol, which are normative for RCTs, emerged as barriers to implementation of ACT within VHA acute psychiatry units. A further perceived barrier was the individual therapy format, which was used in previous ACT RCTs [3, 9].

Relaxing restrictions (e.g., flexible application of the manual) while tailoring the intervention (e.g., offer group format, facilitate buy-in by inpatient staff to engage patients in an ACT-consistent manner) may increase the ability for future studies to build an evidence base to inform decisions regarding broader implementation. While this approach is often promoted in effectiveness trials, it appears similarly important for preliminary studies of psychotherapy on an inpatient unit. It may prove expeditious to utilize Hybrid Type II or III trials that emphasize implementation-related outcomes more than patient-related outcomes [6].

## Abbreviations

ACT: acceptance and commitment therapy
RCT: randomized controlled trial
RE-AIM: reach, efficacy, adoption, implementation and maintenance
TAU: treatment as usual
VHA: Veterans Health Association

## References

[1] American Psychiatric Association (2000). Diagnostic and statistical manual of mental disorders.

[2] Attkisson CC, Zwick R (1982). The client satisfaction questionnaire: psychometric properties and correlations with service utilization and psychotherapy outcome. Eval Progr Plan.

[3] Bach P, Hayes SC (2002). The use of acceptance and commitment therapy to prevent the rehospitalization of psychotic patients: a randomized controlled trial. J Consult Clin Psychol.

[4] Bowersox NW, Visnic S, Valenstein M, McCarthy JF. Care for VHA users with psychosis in the Veterans Health Administration: FY11: 15th annual national psychosis registry report. Serious Mental Illness Treatment Resource and Evaluation Center: Veterans Health Administration; 2013.

[5] Carpenter WT, Kirkpatrick B (1988). The heterogeneity of the long-term course of schizophrenia. Schizophr Bull.

[6] Curran GM, Mukherjee S, Allee E, Owen RR (2008). A process for developing an implementation intervention: QUERI series. Implement Sci..

[7] Dixon LB, Dickerson F, Bellack AS, Bennett M, Dickinson D, Goldberg RW, Peer J (2010). The 2009 schizophrenia PORT psychosocial treatment recommendations and summary statements. Schizophr Bull.

[8] Forman J, Damschroder LJ, Robinson CH, Heisler M, Kerr EA (2010). RE-AIM Plus: expanding the RE-AIM framework for real-time program evaluation.

[9] Gaudiano BA, Herbert JD (2006). Acute treatment of inpatients with psychotic symptoms using acceptance and commitment therapy. Behav Res Ther.

[10] Glasgow RE, Vogt TM, Boles SM (1999). Evaluating the public health impact of health promotion interventions: the RE-AIM framework. Am J Public Health.

[11] Hayes SC, Strosahl KD, Wilson KG (1999). Acceptance and commitment therapy: an experiential approach to behavior change.

[12] Horvath AO, Greenberg LS (1989). Development and validation of the Working Alliance Inventory. J Couns Psychol.

[13] Irmiter CA, McCarthy JF, Barry KL, Soliman S, Blow FC (2007). Reinstitutionalization following psychiatric discharge among VA patients with serious mental illness: a national longitudinal study. Psychiatr Q.

[14] Kovnick JA, Appelbaum PS, Hoge SK, Leadbetter RA (2003). Competence to consent to research among long-stay inpatients with chronic schizophrenia. Psychiatr Services.

[15] McCarthy JF, Blow FC, Valenstein M, Fischer EP, Owen RR, Barry KL, Ignacio RI (2007). VA health system and mental health treatment retention among patients with serious mental illness: evaluating accessibility and availability barriers. Health Serv Res..

[16] Rounsaville BJ, Carroll KM, Onken LS (2001). A stage model of behavioral therapies research: getting started and moving on from stage I. Clin Psychol.

[17] Walker EF, Diforio D (1997). Schizophrenia: a neural diathesis-stress model. Psychol Rev.

